# The Energy Expenditure of Stair Climbing One Step and Two Steps at a Time: Estimations from Measures of Heart Rate

**DOI:** 10.1371/journal.pone.0051213

**Published:** 2012-12-12

**Authors:** Lewis G. Halsey, David A. R. Watkins, Brendan M. Duggan

**Affiliations:** 1 Department of Life Sciences, University of Roehampton, London, United Kingdom; 2 Royal (Dick) School of Veterinary Studies, University of Edinburgh, Edinburgh, United Kingdom; University of Bath, United Kingdom

## Abstract

Stairway climbing provides a ubiquitous and inconspicuous method of burning calories. While typically two strategies are employed for climbing stairs, climbing one stair step per stride or two steps per stride, research to date has not clarified if there are any differences in energy expenditure between them. Fourteen participants took part in two stair climbing trials whereby measures of heart rate were used to estimate energy expenditure during stairway ascent at speeds chosen by the participants. The relationship between rate of oxygen consumption (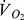) and heart rate was calibrated for each participant using an inclined treadmill. The trials involved climbing up and down a 14.05 m high stairway, either ascending one step per stride or ascending two stair steps per stride. Single-step climbing used 8.5±0.1 kcal min^−1^, whereas double step climbing used 9.2±0.1 kcal min^−1^. These estimations are similar to equivalent measures in all previous studies, which have all directly measured 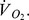 The present study findings indicate that (1) treadmill-calibrated heart rate recordings can be used as a valid alternative to respirometry to ascertain rate of energy expenditure during stair climbing; (2) two step climbing invokes a higher rate of energy expenditure; however, one step climbing is energetically more expensive in total over the entirety of a stairway. Therefore to expend the maximum number of calories when climbing a set of stairs the single-step strategy is better.

## Introduction

The growing problem of obesity in developed countries [1] may in part be due to a lack of opportunity for people to undertake strenuous physical exercise in their daily lives. Reasons include time or money restrictions, or a lack of suitable facilities [2]. Stairs provide a ubiquitous and cost-effective opportunity to incorporate physical exercise into the daily routine. Undertaking regular bouts of physically demanding exercise is known to be beneficial for general health [3]. Indeed, stair climbing has been shown to enhance muscle recruitment and improve cardiovascular capacity [4], [5], [6]. It can also require a noteworthy degree of energy expenditure and recently a number of studies have investigated the energy costs of stair climbing, in part to ascertain the calorie burning value of such exercise. For example, one study [5] calculated the energy cost of ascending stairs one step at a time to be 10.2 kcal min^−1^.

As Gottschall et al. [6] note, there are two practical strategies for stair climbing; ascending stairs one step at a time (contacting each step and with alternating feet per step) and ascending stairs two steps at a time (contacting every other step and with alternating feet per contacted step). However, comparisons to date of the energetic costs of these two strategies are limited. While Aziz and Teh [7] compared the costs of single step ascents with double step ascents, climbing paces were prescribed to control for pace differences between climbing strategies and thus the results are not fully relevant to natural stair climbing situations. Gottschall et al. [6] recorded the energy expenditure of participants walking on an inclined treadmill at their preferred pace, to attempt to simulate single step and double step stair climbing, however as the authors state, clearly there are differences in stair climbing and incline walking, most notably the angle of the ankle during stance.

To our knowledge all previous studies of the energy costs of stair climbing have recorded rate of oxygen consumption (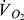) as an indirect measure of rate of energy expenditure. However, there are a number of methods for measuring or estimating energy expenditure [8], some of which are typically cheaper than respirometry. In particular, proxies for energy expenditure such as heart rate and body movement, once calibrated, can be used to ascertain metabolic rate via just a small data logger adorned by the subject [9], [10]. Proxy methods can, therefore, have logistical and financial benefits compared to using a relatively large and expensive portable respirometry system.

**Table 1:** Mean values ±1 Standard Error of the Mean for climbing behaviour and heart rate, and ± Standard Error of the Estimate for energetics variables.

	Ascent duration (s)	Stride rate(steps s^−1^)	Heart rate (beats min^−1^)	Rate of oxygen consumption (mL min^−1^)	Rate of energy expenditure (kcal min^−1^)	Rate of energy expenditure (kcal step^−1^)	Rate of energy expenditure (kcal stride^−1^)	Energy expenditure to ascend 15 m (kcal)
One step	57.3±1.3	1.51±0.04	146.3±5.1	1702.2±14.2	8.5±0.07	0.09±0.01	0.09±0.01	8.62±1.02
Two step	47.1±1.3	0.93±0.03	150.4±5.1	1834.6±16.1	9.2±0.08	0.08±0.01	0.17±0.02	7.59±1.08

Therefore, given that heart rate often correlates linearly and strongly with rate of energy expenditure during activity [11], the present study has two aims: (1) to test the heart rate method as a technique for estimating the energy costs of stair climbing in order (2) to compare the energy costs of one- and two-step stair climbing at natural speeds i.e. those chosen by participants.

**Table 2:** Previous studies measuring or estimating the energy expenditure of stair climbing.

	N	Stair step Pattern	Stair step rate(per min)/Totalascent duration (s)§	Height of stair steps (m)	Mean participant body mass (kg)	Method	Mean energy expenditure during stairway ascending (kcal min^−1^)
O’ Connell et al. (1986) [22]	17	Single	80	0.20	82	Stair treadmill	8.9*
Butts et al. (1993) [23]	28	Single	60, 77, 95, 102	0.20	69	Stepper	13**
Bassett et al. (1997) [24]	18	Single	70	0.20	67	Motorised escalator	10.1
Boreham et al. (2002) [4]	12	Single	88/∼135	0.17	56	Public stairway (Singleascent of 32.8 m)	7.8*
Teh and Aziz (2002) [5]	103	Single	95/116	0.15	61	Public stairway (Singleascent of 27 m)	10.2
Aziz and Teh (2005) [7]	30	Single and double	Single = 100 double = 50/149	0.15	60	Public stairway (Singleascent of 27 m)	Single = 10.4† double = 9.9
Gottschall et al. (2010) [6]	12	Single and double	Single = 109 double = 83/420	n/a	70	Treadmill at 30° incline(Single ascent)	Single = 9.9† double = 11.3
Present study	14	Single and double	Single = 90.6 double = 55.8/52	0.16	64	Public stairway(Multiple ascents anddescents of 14.05m)	Single = 8.5† double = 9.2

* Calculated from 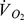 values.

** Estimated from Figure 2 [23]; values at 95 steps per min.

† Statistically significant difference.

§ Where single and double step conditions were undertaken, the value given is the mean across conditions.

## Methods

The methods were approved by the University of Roehampton Ethics Committee. All 14 participants (eight males and six females; mean mass = 64.3±1 SD 8.9 kg) completed a consent form and a PAR-Q questionnaire prior to selection for participation in the study. They subsequently completed a laboratory treadmill protocol to calibrate heart rate with 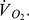 After a rest period of 5 minutes, participants carried out stair climb trials on a public access stairway. Both the treadmill protocol and stair climb trials were conducted at the University of Roehampton. Heart rate was measured during the stair climb trials to estimate 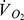 based on the individual-specific 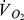 prediction equations. Air temperature ranged between 22 and 24°C.

After completion of a 5 minute warm up on a treadmill (Woodway Ergo ELG 70) at 5 km h^−1^ and 0° incline, the heart rate (Polar CS100 wearlink and transmitter) and 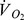 (Jaeger Oxycon Pro) of each participant were measured while they then walked at different gradients (0, 3, 5, 8, 10, 12°), again at 5 k h^−1^. Gradients were experienced in random order. Heart rate and breath by breath 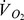 were recorded at 0.2 Hz during the final 30 seconds of each 2 minute gradient stage.

Stair climb trials were completed in a random order on a public access stairway consisting of 86 steps and with a total vertical displacement of 14.05 m (each step averaged 16.3 cm in height) over 8 flights and 5 floors. Each flight was separated by a horizontal connecting platform with a minimum area of 4 m^2^ (representing a negligible proportion of the total cost to climb the stairway [12]). The duration of ascent of the stairway available for the present study was typically less than one minute. This was deemed unlikely to be sufficient time for participants to reach cardio-respiratory steady state [4], [5], [13] and thus the protocol required multiple consecutive stairway ascents. A trial therefore consisted of a total of four ascents interspersed by three descents where participants were accompanied by the investigator to assure compliance with the outlined protocol and to record ascent and descent durations. Participants were fitted with a heart rate monitor, recording at 0.2 Hz, and instructed to ascend and descend at a constant pace of their choice so long as it was not too quick to elicit ‘bounding’. They were instructed not to use handrails or place their hands on their thighs. Two stair climb trials were undertaken; a single step ascent (ascending one stair step per stride) and a double step ascent (ascending two steps per stride). All descents were single step.

### Calculations and Statistical Analyses

**Figure 1:**
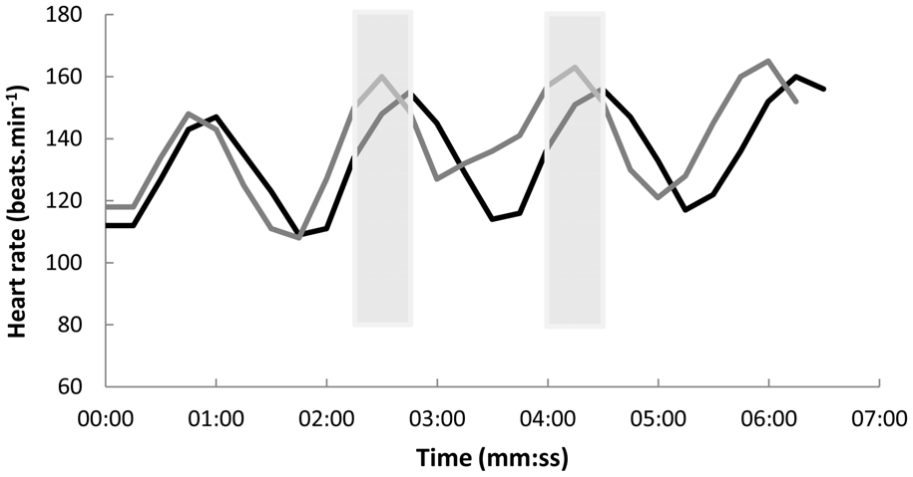
Heart rate over time during four stairway ascents and three descents. The black line represents ascending the stairs one stair step at a time (with alternate feet) while the grey line represents ascending two stair steps at a time. The grey bars represent the final 30 second periods of the second and third ascents of the one step climbing, from which a mean heart rate was obtained to estimate rate of oxygen consumption during stair ascending one stair step at a time. The same method was employed for two step climbing.

The descent required after each ascent could possibly have affected heart rate and 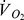 due to different power demands and kinematics [5], [14]. However, it was anticipated that after sufficient ascents steady state would nonetheless be reached during them, indicated by plateaued heart rate, because steady state for a specific activity (in this case stair ascending) is reached more quickly immediately after exercise (in this case stair ascending and descending) [13]. In turn, the final 30 s of some stairway ascents were anticipated to be periods during which estimated 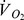 represented rate of energy expenditure. Inspection of the heart rate data per individual subsequent to data collection suggested that heart rate was at least approximately plateauing towards the end of each stairway ascent beyond the first ascent, although peak values were slightly higher on the fourth ascent compared to the second and third (Figure 1). This pattern fitted with the likelihood that steady state was not reached during the first ascent and that some participants reported or exhibited fatigue by the fourth ascent. Therefore, mean heart rate during the final 30 s of the second and third ascents was calculated for each participant to estimate 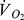, at an individual level, during stairway ascent. Estimated 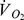 was converted to rate of energy expenditure (kcal min^−1^) assuming 1 L O_2_ = 5 kcal [15].

Such that the present calibration data can be used in future studies for estimating 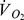 from new measures of heart rate during stair climbing or related activities, a mixed linear model (SPSS, IBM, USA), including participant identification as a random factor to account for non-independence of the data, was generated to produce a 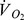 prediction equation from heart rate.

Descriptive statistics are provided as mean values±1 SE. Means of estimates, i.e. 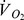 and all values derived from this (estimated energy expended per stair step ascended, estimated energy expended per stride while ascending, estimated energy expended to ascend a flight of stairs 15 m in height), are calculated accounting for differences in the errors associated with the estimate for each participant; each individual value included in the calculation is weighted by the reciprocal of the associated variance. Likewise, the SEs of the estimate means are adjusted. Consequently the SE of estimate means are smaller than would be calculated for unweighted means since in the former those individual values with less error have more influence on the mean calculation.

Paired t tests, calculated in Excel (Microsoft Corp., USA), were used to compare the measured variables (ascent duration, stride stepping rate, heart rate) between the two trials. Proximate normal z tests for paired comparisons, again calculated in Excel, compared all estimated values, since they account for the estimate errors. Proximate normal z tests indicate strong evidence for a difference if z>|1.96|, i.e. P<0.05. For estimate metrics derived from 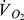 the associated variance for each individual value was calculated by taking the variance associated with the equivalent estimated 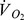 value and adjusting it proportionately to the difference between that estimated 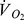 value and the derived estimate value, i.e. the variance values were isometrically scaled to the change in mean values.

## Results

Table 1 summarises the measures recorded in the present study. Kolmogorov-Smirnov tests provided no evidence that any of the variables were non-normally distributed. Ascent duration was significantly lower for the two step ascent (t_13_ = 10.62, p<0.001), as was stride rate (t_13_ = 25.89, p<0.001). The linear equation to estimate 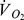 from heart rate during stair climbing and similar activities, based on the data of the present study, is:
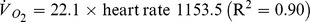



Mean heart rate during periods of steady state ascent was lower for the one step ascent (t_13_ = 2.63, p = 0.021), as was estimated 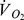 during those periods (z = 2.18). Estimated rate of energy expenditure during stairway ascent was 8.5±0.1 kcal min^−1^ during the one step ascent and 9.2±0.1 kcal min^−1^ during the two step ascent. Rate of energy expenditure per stride was higher (z = 2.90, p<0.05) during the two step ascent while there was no significant difference in rate of energy expenditure per stair step (z = 0.64, p>0.05). The total energy expended to climb a stairway 15 m in height with one step ascent is greater than with two step ascent (z = 6.150, p<0.05).

Due to the variation in the relationship between 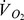 and heart rate between individuals, heart-rate based prediction equations can only be used to predict heart rate in new individuals at the group level. That is, the above equation can only reasonably provide an estimate of 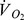 for the mean of a group of individuals (typically at least six) [16]. A measure of error associated with an estimate of mean 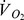 derived from the prediction equation must account for the error inherent in the estimate (standard error of the estimate; SEE). To calculate this, information is required that is derived from the raw data and the generation of the above equation. These data are provided here for putative future studies that apply the prediction equation presented in the current study: number of individuals in the model to generate the equation (n_1_) = 14, number of data points in the model to generate the equation (n_2_) = 86, variance estimate of participant identification as a random factor (d^2^) = 146435.7, variance estimate of the error (e^2^) = 23851.1, the mean value of heart rate used in the regression (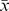) = 117.8 and the sum of the squares of all the values of heart rate less mean heart rate (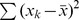), (termed 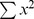 in [16]), = 39121.3. For details of calculating an SEE for mean estimated 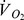, refer to [16].

## Discussion

The present data show that climbing flights of stairs two steps at a time requires a higher rate of energy expenditure than climbing them one step at a time. A similar finding was reported by Gottschall et al. [6] for treadmill walking on a representative incline at voluntarily selected paces for one- and two-step stair climbing. However, no previous studies to our knowledge have reported that *total* energy expenditure is lower for ascending a stairway two steps at a time; the higher *rate* of energy expenditure when ascending two at a time is due to the higher preferred pace of participants in this situation (see Aziz and Teh [7]).

Rates of energy expenditure of stairway ascending reported in the current study are very similar to those provided by previous studies investigating the cost of stair climbing or similar climbing scenarios (Table 2). This indicates that the use of heart rate as a proxy for rate of energy expenditure during stair climbing, where heart rate has been calibrated using an inclined treadmill, is an accurate method of estimation, despite these two activities being somewhat different (and even when the experimental design involves periods of stairway descent punctuating the periods of ascent). In turn, this perhaps indicates that incline treadmill walking and stairway ascending have similar energy costs and evoke similar muscle utilization. The underlying explanation for this could be that the overriding cost involved with both stair ascending and walking up an equivalent incline is that required to raise the centre of mass against gravity [12], [17].

Previous work has measured 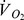 using respirometry and this requires expensive portable gas analysis equipment or Douglas bags, which can be cumbersome for measuring activity. Furthermore, there have been reports that wearing a mask can be considered restrictive to breathing during periods of high respiratory activity [18], [19] and some participants experience restricted vision. In contrast, measuring heart rate requires only a cheap and relatively simple heart rate monitor. Future studies using heart rate as a proxy for rate of energy expenditure during stair climbing or related activities can use the prediction equation provided in the present study for groups of participants that are healthy adults (and approximately within the range of masses of the participants in the present study). Proponents are reminded that estimates of energy expenditure obtained this way will have greater associated errors than measures of energy expenditure obtained through e.g. respirometry because of the imperfect fit of the 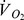
–heart rate calibration data.

The power required to lift the body against gravity during stair climbing explains the greater energy expenditure per stride during two step ascents; the body is lifted twice the height per stride [14]. However, total energy expenditure to ascend a stairway of typical height (15 m) is greater during one step ascents (although the present study did not find a difference in energy cost per stairway step between one and two step ascents this is likely due to sensitivity limitations in predicting 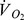 from heart rate for very short periods of time). The greater total energy expenditure of one step ascents of stairways is presumably explained at least in part by the greater ascent duration. However, there may also be a biomechanical explanation as well. Since stair step rate is higher during single stepping this may result in faster rates of muscle shortening, which increases energy turnover [20], and the greater recruitment of fast twitch muscle fibres which are less economical. See Aziz and Teh [7] for a detailed commentary on biomechanical explanations for heightened energetic costs associated with stepping faster.

The advice to those seeking to utilise stair climbing specifically as a method to control or reduce weight [21] is to ascend stairways one step at a time; more calories are burned through this form of stair climbing. For example, climbing just a 15 m high stairway five times a day represents an energy expenditure of on average 302 kcal per week using the one step strategy and 266 kcal using the two step strategy.
