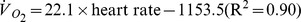# Correction: The Energy Expenditure of Stair Climbing One Step and Two Steps at a Time: Estimations from Measures of Heart Rate

**DOI:** 10.1371/journal.pone.0100658

**Published:** 2014-06-13

**Authors:** The PLOS ONE Staff

There is an error in the linear equation presented in the Results section. A subtraction sign was omitted during the typesetting process. Please see the corrected equation below.